# RCD1 Promotes Salt Stress Tolerance in *Arabidopsis* by Repressing ANAC017 Activity

**DOI:** 10.3390/ijms24129793

**Published:** 2023-06-06

**Authors:** Jinyuan Tao, Feiyan Wu, Haoming Wen, Xiaoqin Liu, Weigui Luo, Lei Gao, Zhonghao Jiang, Beixin Mo, Xuemei Chen, Wenwen Kong, Yu Yu

**Affiliations:** 1 Guangdong Provincial Key Laboratory for Plant Epigenetics, Longhua Bioindustry and Innovation Research Institute, College of Life Sciences and Oceanography, Shenzhen University, Shenzhen 518060, China; 2 Key Laboratory of Optoelectronic Devices and Systems of Ministry of Education and Guangdong Province, College of Optoelectronic Engineering, Shenzhen University, Shenzhen 518060, China; 3 Institute of Advanced Agricultural Science, Peking University, Weifang 261000, China; 4 Department of Botany and Plant Sciences, Institute for Integrative Genome Biology, University of California, Riverside, CA 92521, USA

**Keywords:** salt response, transcription inhibition, translocation, SOD activity

## Abstract

Plants have evolved diverse strategies to accommodate saline environments. More insights into the knowledge of salt stress regulatory pathways will benefit crop breeding. RADICAL-INDUCED CELL DEATH 1 (RCD1) was previously identified as an essential player in salt stress response. However, the underlying mechanism remains elusive. Here, we unraveled that *Arabidopsis* NAC domain-containing protein 17 (ANAC017) acts downstream of RCD1 in salt stress response, and its ER-to-nucleus transport is triggered by high salinity. Genetic and biochemical evidence showed that RCD1 interacts with transmembrane motif-truncated ANAC017 in the nucleus and represses its transcriptional activity. Transcriptome analysis revealed that genes associated with oxidation reduction process and response to salt stress are similarly dysregulated in loss-of-function *rcd1* and gain-of-function *anac017-2* mutants. In addition, we found that ANAC017 plays a negative role in salt stress response by impairing the superoxide dismutase (SOD) enzyme activity. Taken together, our study uncovered that RCD1 promotes salt stress response and maintains ROS homeostasis by inhibiting ANAC017 activity.

## 1. Introduction

High salinity stress severely impacts the growth and development of plants throughout the life cycle, leading to yield loss for major food crops [1,2,3]. As sessile organisms, plants have evolved various strategies to cope with high salinity stress. In the past decades, numerous studies have gradually illustrated the complex regulatory network of salinity stress responses in plants, as well as the interplay of salt stress response and other signaling pathways, such as phytohormones signaling [2,4,5].

In *Arabidopsis*, the RADICAL-INDUCED CELL DEATH 1 (RCD1) protein was characterized as an important cellular hub for different stress responses, phytohormone signaling pathways, and plant growth and development. A mutant of *RCD1* was first isolated based on its sensitivity to apoplastic reactive oxygen species (ROS), particularly superoxide [6], and loss-of-function *rcd1* mutants exhibit developmental defects, including dwarfed stature, altered leaf and rosette morphology, increased branching, early flowering, and early senescence [6,7]. Subsequent studies reported the functions of RCD1 in response to various stresses and several phytohormones, including salinity, UV-B, heat, high light, ethylene, abscisic acid (ABA), and methyl jasmonate [7,8,9,10]. These pleiotropic effects of RCD1 have been attributed to its diverse interacting partners, including many transcription factors (TFs) involved in different regulatory pathways, via the C-terminal RCD1-SRO1-TAF4 (RST) domain [11,12]. Intriguingly, the RCD1 protein is predominantly localized in the nucleus under unstressed conditions, and the nuclear localization is conferred by three N-terminal nuclear localization signals (NLSs) with the assistance of the WWE and poly ADP-ribose polymerase (PARP) domains. Under salt stress conditions, RCD1 is also present in the cytoplasm, where it interacts with the plasma membrane-localized Na^+^/H^+^ antiporter SALT OVERLY SENSITIVE 1 (SOS1) to respond to salt stress [9,13,14]. Moreover, *Aribidopsis* plants overexpressing the full-length *RCD1* exhibit more resistance to salt stress [12].

Recently, RCD1 was identified as a hub to integrate retrograde signaling from chloroplast and mitochondria through two TFs, *Arabidopsis* NAC domain-containing proteins 13 and 17 (ANAC013 and ANAC017) [15]. ANAC017 is a master positive effector in the retrograde signaling network and is anchored on the endoplasmic reticulum (ER) membrane through its C-terminal transmembrane™ domain under normal conditions. When retrograde signaling is triggered, such as when electron transfer in chloroplasts and mitochondria is compromised by methyl viologen (MV) and antimycin A (AA) treatment, respectively, ANAC017 is cleaved at the TM, and its N-terminal fragment enters the nucleus to directly activate the expression of numerous mitochondrial dysfunction stimulon (MDS) genes, including its homolog *ANAC013* and an indicator gene of mitochondrial retrograde signaling *ALTERNATIVE OXIDASE 1a* (*AOX1a*) [16,17,18,19]. Notably, overexpression of *ANAC017* resulted in retarded growth similar to the *rcd1* mutant, and this was attributed to the abnormal expression of MDS genes activated by ANAC017 [19], as loss-of-function of *ANAC017* in *rcd1* could rescue the plant phenotype [15]. These findings suggest that ANAC017 may act as a major downstream factor of RCD1 to modulate plant growth and development through retrograde signaling. However, it remains unknown whether ANAC017 participates in RCD1-mediated salt stress response.

ROS are byproducts of various metabolic processes, including superoxide anion (O_2_^•−^), hydrogen peroxide (H_2_O_2_), singlet oxygen (^1^O_2_), and hydroxyl radical (OH^•^) [20,21,22]. ROS are distributed widely in plant cells at low levels and act as signaling molecules to participate in many developmental processes. In retrograde signaling from both chloroplasts and mitochondria, ROS are thought to be intracellular signals and induce the expression of nuclear genes in an ANAC017-dependent manner [17,23,24]. On the other hand, a ROS burst serves as an indicator of salt stress-induced early response [3,4,25]. The production of ROS is greatly induced by high salinity, and over-accumulated ROS result in damage to cell structure and functions and activation of the antioxidant scavenging system [26,27]. Superoxide dismutase (SOD), catalase (CAT), and peroxidase (POD) are major enzymes in the enzymatic antioxidant system to maintain ROS homeostasis. After exposure to salt stress, chloroplast- and/or mitochondria-produced O_2_^•−^ is quickly converted into H_2_O_2_ by SOD, followed by the reduction of H_2_O_2_ to water through CAT, POD, and other reductases [28,29].

In this study, we demonstrated a novel role of ANAC017 in salt stress response. Similar to AA treatment, high salinity triggered the translocation of ANAC017 from the ER membrane to the nucleus, where it negatively regulates salt stress tolerance by affecting ROS scavenging enzyme activities to disrupt ROS homeostasis. In the nucleus, RCD1 interacted with ANAC017 to repress its transcriptional activity, thus maintaining ROS homeostasis and enabling salt stress responses.

## 2. Results

### 2.1. ANAC017 Participates in RCD1-Mediated Salt Stress Response

**Figure 1:**
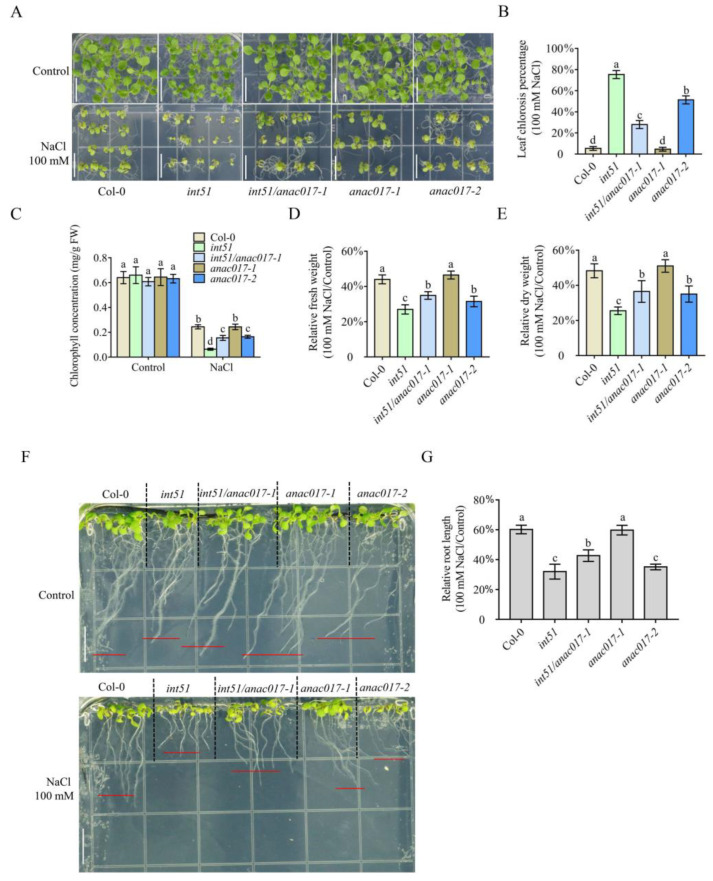
Loss of function in *ANAC017* partially rescues the salt-sensitive phenotype of *int51*. (**A**) The phenotype of fourteen-day-old Col-0, *int51*, *int51/anac017-1*, *anac017-1,* and *anac017-2* (lacking the transmembrane motif) seedlings grown on 1/2 MS medium with or without 100 mM NaCl treatment. Bar scale, 1 cm. (**B**) Quantitative analysis of leaf chlorosis percentage in WT and mutants under treatment with 100 mM NaCl. Data were collected from five biological replicates and presented as mean ± standard deviation. Fifty seedlings were used for each replicate. Letters indicate significant differences at *p*-value < 0.05 based on one-way ANOVA and Tukey’s HSD test. (**C**) Quantitative analysis of chlorophyll concentration in WT and mutants. Data were collected from five biological replicates and presented as mean ± standard deviation. Around 0.1 g seedlings were used for each replicate. Letters indicate significant differences at *p*-value < 0.05 based on one-way ANOVA and Tukey’s HSD test. FW, fresh weight. (**D**,**E**) Quantitative analysis of the relative fresh weights (**D**) and relative dry weights (**E**) (100 mM NaCl/Control) in WT and mutants. Data were collected from five biological replicates and presented as mean ± standard deviation. Five seedlings were used for each replicate. Letters indicate significant differences at *p*-value < 0.05 based on one-way ANOVA and Tukey’s HSD test. (**F**) The root phenotypes of seven-day-old Col-0, *int51*, *int51/anac017-1*, *anac017-1*, and *anac017-2* seedlings vertically grown on 1/2 MS medium with or without 100 mM NaCl treatment. Bar scale, 1 cm. (**G**) Quantitative analysis of the relative root lengths (100 mM NaCl/Control) in WT and mutants. Data were collected from fifteen–twenty plants per genotype and presented as mean ± standard deviation. Letters indicate significant differences at *p*-value < 0.05 based on one-way ANOVA and Tukey’s HSD test.

A previous study revealed that RCD1 coordinates chloroplast and mitochondrial retrograde signaling through ANAC017, and the leaf phenotypes of *rcd1* were mostly rescued in the *rcd1/anac017* double mutant [15], suggesting that ANAC017 integrates RCD1-regulated retrograde signaling and plant growth and development pathways. To determine whether ANAC017 also plays a pivotal role in RCD1-mediated salt stress response, wild type (WT) Col-0 and four mutants, including *int51* (a mutant in *RCD1*), *anac017-1* (a loss-of-function mutant), *int51/anac017-1*, and *anac017-2* (a gain-of-function mutant lacking the TM motif), were treated with high salinity. As compared with the corresponding plants grown under normal conditions, the fresh weights of Col-0 and *anac017-1* did not change under 80 mM salt, but the fresh weight and dry weight of *int51* were significantly decreased under 80 mM salt (Figure S1), which was consistent with the previous report that an *rcd1* mutant was more sensitive to salt treatment than WT [9]. Similarly, the fresh weight and dry weight of *anac017-2* were also greatly reduced (Figure S1). Notably, the fresh weights and dry weights of the *int51/anac017-1* double mutant were comparable between normal conditions and 80 mM salt stress (Figure S1), indicating that ANAC017 may act downstream of RCD1 in salt stress response. Next, we performed treatments with 100 mM salt. The *int51* mutant exhibited significantly increased leaf chlorosis percentage, meanwhile substantially reduced plant size and chlorophyll content, as well as relatively decreased fresh weight and dry weight, and short root length, under salt treatment as compared to WT (Figure 1), suggesting its higher sensitivity to salt than WT. Consistently, *anac017-2* also showed higher sensitivity to salt stress in comparison to WT, as indicated by the remarkably increased leaf chlorosis percentage and reduced plant size, chlorophyll content, decreased fresh weight and dry weight, and short root length (Figure 1). Both the *anac017-1* single mutant and the *int51/anac017-1* double mutant displayed comparable plant size to WT under the salt condition, but the increased leaf chlorosis percentage and the reduced chlorophyll content, decreased fresh weight and dry weight, and short root length in *int51* were only partially rescued in the *int51/anac017-1* double mutant (Figure 1B–E,G). These genetic observations implied that ANAC017 acted downstream of RCD1 to play a negative role in regulating salt stress response.

### 2.2. ANAC017 Translocates from the ER to the Nucleus in Response to Salt Stress

**Figure 2:**
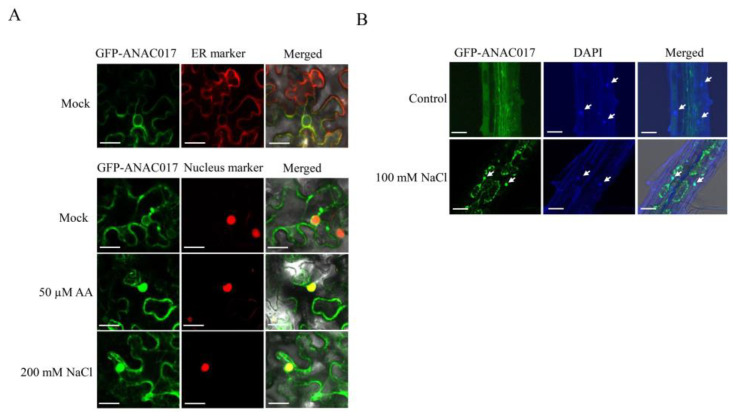
Salt stress triggers the translocation of ANAC017 from the ER to the nucleus. (**A**) The RFP-fused ER marker and mCherry-fused nucleus marker were separately co-infiltrated into the five-week-old *N. benthamiana* leaves with the plasmid of N-terminal GFP-fused full-length *ANAC017*, and the plants were grown for three days under normal conditions. Then the plants were treated with 50 µM AA (antimycin A) or 200 mM NaCl for 3 h before the GFP and RFP signals were observed using a confocal laser scanning microscope. The leaves without treatment served as the mock control. Two independent experiments were performed. ER marker, ER-rk *CD3-959*. Nucleus marker, H2B-mCherry. Bar scale, 25 µm. (**B**) The roots of seven-day-old *35S::GFP-ANAC017* transgenic plants harboring the fused GFP-ANAC017 protein were treated with or without 100 mM NaCl for 6 h, and the GFP and 4′,6-diamidino-2-phenylindole (DAPI) staining were observed using a confocal laser scanning microscope. White arrows indicated the cell nuclei. Two independent experiments were performed. Bar scale, 25 µm.

ANAC017 translocates from the ER to the nucleus to activate the expression of MDS genes in retrograde signaling, as evidenced by AA treatment of onion cells [17]. To examine whether high salinity triggers the translocation of ANAC017, we fused the green fluorescent protein (GFP) to the N-terminus of ANAC017 and observed the subcellular localization of GFP-ANAC017 with or without salt treatment. The C-terminal GFP-fused RCD1 (RCD1-GFP) served as a control for nuclear localization. The results showed that transiently expressed GFP-ANAC017 and RCD1-GFP in *Arabidopsis* protoplasts without treatment were colocalized with the ER marker and the nuclear marker, respectively (Figure 2, Supplemental Figure S2A,B). To better observe the subcellular locations of ANAC017, we next transiently expressed GFP-ANAC017 in *Nicotiana benthamiana* leaves. As expected, GFP-ANAC017 originally localized to the ER and encircled the nucleus and subsequently relocated to the nucleus when leaves were subjected to AA treatment (Figure 2A), which was consistent with observations in onion epidermal cells [17]. Similar results were observed upon salt stress treatment—a portion of GFP-ANAC017 signals was enriched in the nucleus and colocalized with the nucleus marker (Figure 2A). Likewise, we generated and obtained several stable transgenic plants harboring fused GFP-ANAC017 protein and found GFP-ANAC017 signal was enriched substantially in the nuclei of root cells under salt stress treatment (Figure 2B). These results demonstrated that ANAC017 was released from the ER to enter the nucleus in response to salt stress.

### 2.3. Transcriptional Activity of ANAC017 Is Inhibited by RCD1 in the Nucleus

**Figure 3:**
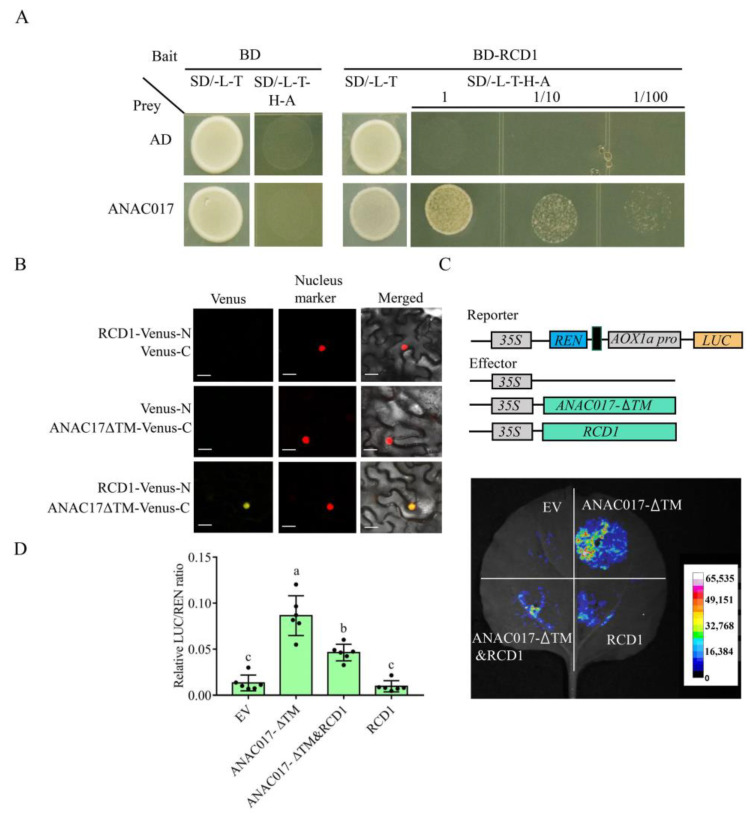
RCD1 interacts with ANAC017 in the nucleus and represses the transcriptional activity of ANAC017. (**A**) The interaction between RCD1 and ANAC017 in the yeast two-hybrid assay. SD/-L-T indicated the synthetically defined medium lacking the amino acids of Leu and Trp. SD/-L-T-H-A indicated the synthetically defined medium lacking the amino acids of Leu, Trp, His, and Ade. (**B**) Bimolecular fluorescence complementation (BiFC) assay between RCD1 and ANAC017ΔTM in *N. benthamiana* leaves. The Venus signal was observed using a confocal laser scanning microscope. Venus-N, N-terminal half of the Venus fluorescent protein, Venus-C, C-terminal half of the Venus fluorescent protein. Experiments were repeated twice. H2B-mCherry served as the nucleus marker. Bar scale, 25 μm. (**C**) Plasmids used in a reporter gene assay are shown in the top panel. The bottom panel shows the activation of the 2 kb *AOX1a* promoter by ANAC017ΔTM and the antagonistic effects of RCD1 towards ANAC017ΔTM. The colors indicate relative expression values. EV: Empty vector. REN: Renilla luciferase. LUC: Luciferase. (**D**) LUC/REN ratios in *N. benthamiana* leaves. Data were collected from six biological replicates and presented as mean ± standard deviation. Letters indicate significant differences at *p*-value < 0.05 based on one-way ANOVA and Tukey’s HSD test.

ANAC017 and RCD1 were reported to interact with each other. However, these two proteins existed in different subcellular locations unless some stresses were imposed. Since ANAC017 is a TF, we speculate that the interaction between RCD1 and ANAC017 takes place in the nucleus. To test this, we first performed the yeast two-hybrid assay, the result showed that RCD1 could interact with ANAC017 in the yeast system (Figure 3A). We also conducted the bimolecular fluorescence complementation (BiFC) experiment with a truncated ANAC017 protein lacking the TM (mimicking *anac017-2*, referred to as ANAC017-ΔTM) fused with the C-terminus of the Venus fluorescent protein and RCD1 tagged with the N-terminus of Venus. When these two proteins were co-expressed in *N. benthamiana* leaves, the Venus signal was present and colocalized with the nuclear marker (Figure 3B). These results indicated that the two proteins interacted in the nucleus. Next, we investigated the effect of RCD1 on the transcriptional activity of ANAC017 using the Luciferase (LUC) reporter system and transient expression in *N. benthamiana* leaves. The effectors were 35S::ANAC017-ΔTM and 35S::RCD1, and the reporter was *LUC* driven by the *AOX1a* promoter (Figure 3B). The results showed that ANAC017-ΔTM was capable of activating *LUC* driven by the *AOX1a* promoter, whereas the activity of ANAC017-ΔTM was markedly reduced when RCD1 was present (Figure 3C,D). These results demonstrated that RCD1 interacted with ANAC017 and inhibited its activity.

**Figure 4:**
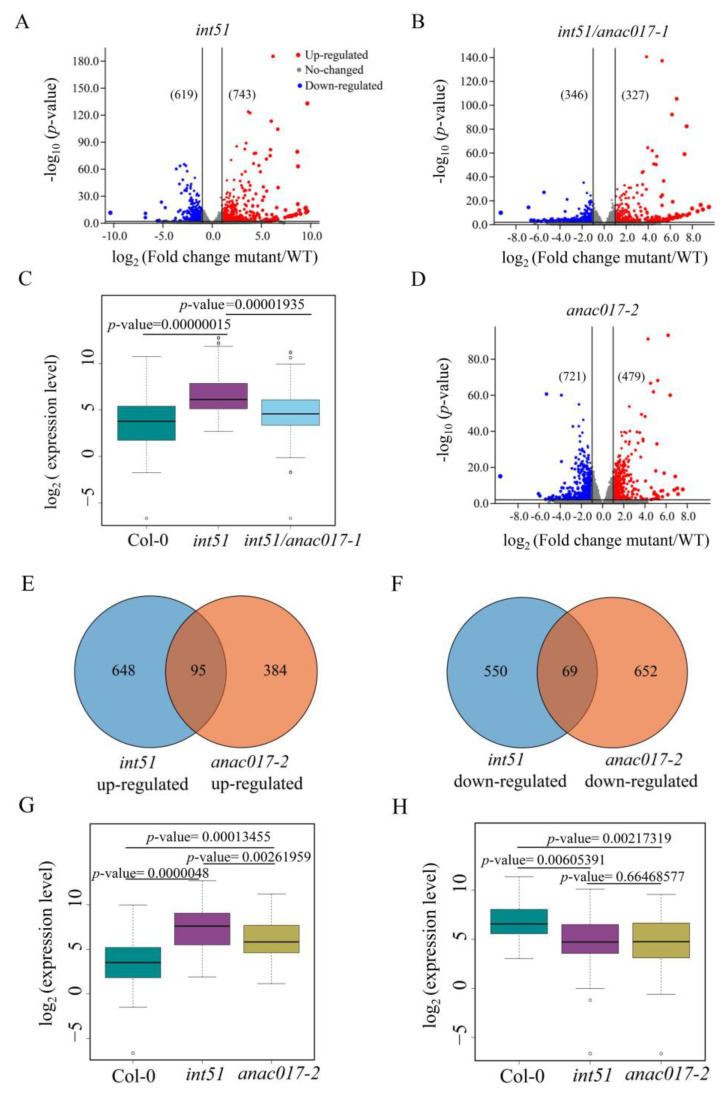
RNA-seq analysis of *int51*, *int51/anac017-1* and *anac017-2*. (**A**,**B**,**D**) Volcano plots representing DEGs in *int51* (**A**), *int51/anac017-1* (**B**) and *anac017-2* (**D**) compared with Col-0. The red, blue, and grey dots represent the up-regulated, down-regulated, and unchanged genes, respectively. The numbers in brackets indicate the numbers of DEGs. (**C**) Box plot presenting the expression levels of 282 genes that are up-regulated genes in *int51* as compared to Col-0 and down-regulated in *int51/anac017-1* as compared to *int51*. (**E**,**F**) Venn diagram showing the overlap in up-regulated genes (**E**) and down-regulated genes (**F**) between *int51* and *anac017-2*. (**G**) Box plot presenting the expression levels of the 95 commonly up-regulated genes in (**E**). (**H**) Box plot showing the expression levels of the 69 commonly down-regulated genes in (**F**).

To examine the genome-wide repression of ANAC017 activity by RCD1, we performed RNA-seq analysis with 14-day-old seedlings of WT and the *int51*, *int51/anac017-1*, and *anac017-2* mutants. Differentially expressed genes (DEG) in each mutant were determined with the criteria |log2(fold-change)| > 1 and *p*-value < 0.05 when compared with Col-0. In the *int51* mutant, 743 genes were up-regulated, and 649 genes were down-regulated (Figure 4A), while in the *int51/anac017-1* double mutant, 327 up-regulated and 346 down-regulated genes were identified (Figure 4B). Further analysis revealed that 282 of the 743 genes showed significantly decreased expression with the criteria |log2(fold-change)| > 1 and *p*-value < 0.05 in *int51/anac017-1* vs. *int51*, indicating that ANAC017 was responsible for the upregulation of the 282 genes in the *rcd1* mutant (Figure 4C, Supplemental Data Set S1). To explore the potential direct targets of ANAC017, we searched for the mitochondrial dysfunction motif (MDM) (CTTGNNNNNCA[A/C]G), which was reported to be the ANAC017 binding site [17,30], within the promoters (2 kb upstream of transcription start site) and 5′ untranslated regions of the 282 genes, and identified 82 candidate genes that could be regulated by RCD1-ANAC017 directly, including the retrograde signaling reporter genes *AOX1a* and *UGT74E2* (Supplemental Figure S3, Supplemental Data Set S2) [17]. In the gain-of-function *anac017-2* mutant, there were 479 up-regulated and 721 down-regulated genes (Figure 4D). Overlap analysis revealed that 95 up-regulated genes and 69 down-regulated genes were affected in both *int51* and *anac017-2* (Figure 4E,F). The expression levels of the 69 genes in *int51* and *anac017-2* were comparable, whereas the expression levels of the 95 genes in *anac017-2* were a little bit lower than those in *int51* (Figure 4G,H), presumably due to the presence of RCD1, which still repressed ANAC017 activity in *anac017-2*. Taken together, these results suggested that RCD1 interacted with ANAC017 in the nucleus and repressed the transcriptional activity of ANAC017.

### 2.4. Targets of ANAC017-RCD1 Are Involved in the Oxidation-Reduction Process and Salt Stress Response

**Figure 5:**
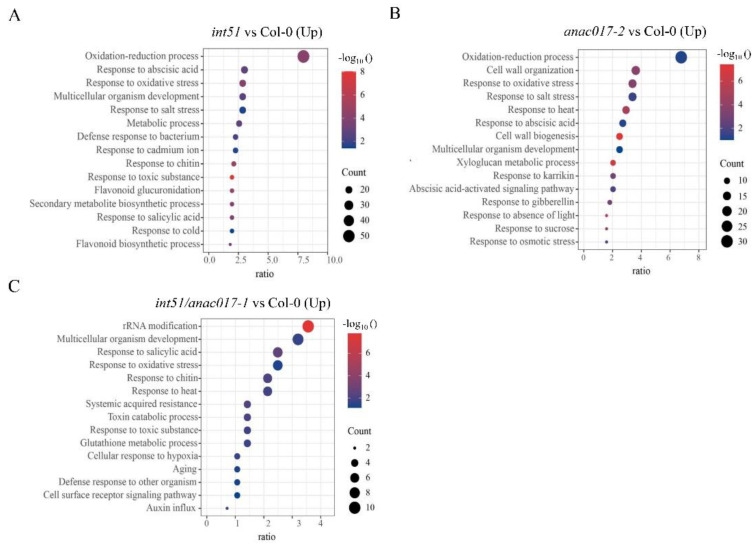
GO enrichment analysis for differentially expressed genes in *int51*, *anac017-2*, and *int51/anac017-1*. (**A**–**C**) GO enrichment analysis of the up-regulated genes in *int51* (**A**), *anac017-2* (**B**), and *int51/anac017-1* (**C**), respectively, compared to Col-0.

To investigate the downstream genes associated with salt stress response, we performed Gene Ontology (GO) enrichment analysis, with a focus on *int51* and *anac017-2* mutants. For up-regulated DEGs in *int51* and *anac017-2*, we found GO terms associated with the oxidation-reduction process and response to salt stress (Figure 5A,B). Notably, both the “oxidation-reduction process” and “response to salt stress” GO terms disappeared in the up-regulated DEGs in the *int51/anac017-1* double mutant (Figure 5C). Similarly, GO terms associated with either oxidation-reduction process or response to salt stress were not enriched in the up-regulated DEGs in *anac017-1* (Supplemental Figure S4A). These results suggested that *ANAC017* promoted the expression of genes involved in salt stress responses and was responsible for the up-regulation of salt-stress-responsive genes in the *int51* mutant.

Among the up-regulated genes in *int51*, we noticed 13 genes belonging to the *ANAC* family. *ANAC013* was reported as a functional homolog of *ANAC017* [16]. The normalized transcript level of *ANAC013* was elevated by more than 10-fold in *int51* compared to Col-0 and was markedly reduced in the *int51/anac017-1* double mutant when compared with *int51* (Supplemental Figure S4B), indicating that the up-regulation of *ANAC013* in *int51* was mostly dependent on ANAC017. In addition, other *ANAC* genes, such as *ANAC03*, *ANAC010*, *ANAC042*, *ANAC044*, *ANAC047*, *ANAC048*, *ANAC074*, *ANAC083*, *ANAC085*, *ANAC087*, and *ANAC103* were also up-regulated in *int51* compared to Col-0, while the extent of the up-regulation was reduced in *int51/anac017-1* except for *ANAC016, ANAC044*, and *ANAC103* (Supplemental Figure S4B). However, no significant difference was found for the transcript abundance of *ANAC017* between *int51* and Col-0, suggesting that RCD1 regulated the expression of ANAC017 post-transcriptionally or the activity of ANAC017 at the protein level. These results indicated that ANAC017 possibly regulates salt stress response by triggering the expression of other *ANAC* genes, which further strengthened the function of ANAC017 as a master regulator.

### 2.5. ANAC017 Acts Downstream of RCD1 to Disrupt ROS Homeostasis through Affecting ROS Scavenging Enzyme Activities

**Figure 6:**
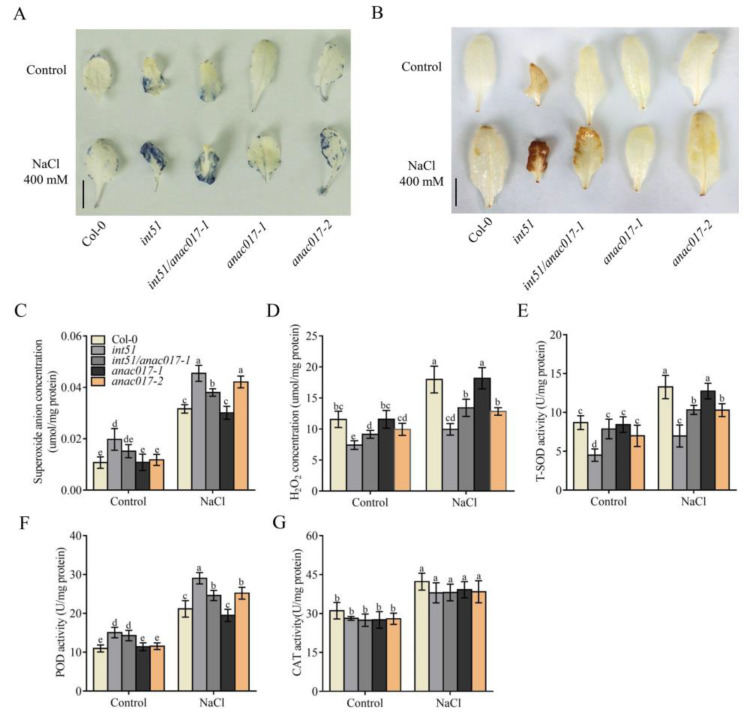
ANAC017 affects ROS homeostasis. (**A**,**B**) NBT staining (**A**) and DAB staining (**B**) in the leaves of Col-0 and mutants (*int51*, *int51/anac017-1*, *anac017-1*, and *anac017-2*). Seven-day-old seedlings grown on 1/2 MS medium were transferred to soil and grown for another two weeks before the treatment with or without 400 mM NaCl for 24 h. Bar scale, 0.5 cm. (**C**–**G**) Quantitative analysis of ROS molecules O_2_^•−^ (**C**) and H_2_O_2_ (**D**) and ROS-related enzyme activities of total SOD dismutase (**E**), POD (**F**), and CAT (**G**) in leaves of Col-0 and mutants. Seven-day-old seedlings grown on 1/2 MS medium were transferred to soil and grown for another two weeks before the treatment with or without 400 mM NaCl for four days. Data were collected from five biological replicates and presented as mean ± standard deviation. About 0.1 g rosette leaves were used in each replicate. Letters indicate significant differences at *p*-value < 0.05 based on one-way ANOVA and Tukey’s HSD test.

High salinity triggers the production of ROS, resulting in oxidative stress in plants [31,32]. According to the GO enrichment analysis, we speculated that ROS homeostasis was disrupted in *int51* and *anac017-2* mutants. To understand the hypersensitivity of *int51* and *anac017-2* to salt stress, we detected the accumulation of ROS (O_2_^•−^ and H_2_O_2_) and the activities of ROS-related enzymes (total SOD dismutase, POD, and CAT) in each genotype with or without salt stress treatment. Nitro blue tetrazolium (NBT) staining showed that O_2_^•−^ accumulated at a higher level in the leaves of *int51* than that of Col-0 under both normal and salt stress conditions (Figure 6A), which was in agreement with previous reports [33,34]. Under salt stress, the accumulation of O_2_^•−^ in the leaves of *int51/anac017-1* was also higher than that of Col-0 but lower than that of *int51* (Figure 6A). Notably, the production of O_2_^•−^ in the leaves of *anac017-2* was substantially increased as compared to Col-0, although there was no change under normal conditions (Figure 6A). The O_2_^•−^ level in the leaves of *anac017-1* was similar to that of Col-0 under both conditions. These results were further confirmed by quantitative measurements of O_2_^•−^ (Figure 6C).

DAB (3,3′-Diaminobenzidine) staining was performed to measure the accumulation of H_2_O_2_. The results were similar to those of NBT staining in that the *int51* mutant leaves showed more stains than wild type under both normal and salt stress conditions, and stronger stains were partially rescued by the *anac017-1* mutation (Figure 6B). However, quantitative measurements of H_2_O_2_ in these mutants gave results contradictory to those of DAB staining (Figure 6D). H_2_O_2_ accumulation in the leaves of *int51*, *int51/anac017-1,* and *anac017-2* was significantly reduced compared to Col-0 under both conditions, except for *anac017-2* under normal conditions. Although the H_2_O_2_ levels in leaves of *anac017-1* were comparable to Col-0 with or without salt stress, those of *int51/anac017-1* were higher than *int51* under both conditions. In DAB staining, the brown color represents the oxidized form of DAB, which is attributed to two factors: H_2_O_2_ and peroxidase. In our RNA-seq, several genes of the peroxidase superfamily showed increased expression levels in these mutants compared to Col-0. Specifically, the transcript levels of 6 genes, including *PRXCB (PEROXIDASE CB*), *DOX1* (*DIOXYGENASE 1*), *PRX52* (*PEROXIDASE 52), AT5G58400*, *AT5G58390*, and *AT2G43480*, were increased in *int51*. In *int51/anac017-1*, *DOX1*, *AT5G58400*, and *AT2G43480* showed elevated expression levels. In *anac017-2*, the transcript levels of *AT4G37530* and *AT5G39580* were increased (Supplemental Figure S5A). These results were in line with quantitative measurements of POD activity under the normal condition except for *anac017-2* (Figure 6G). After salt stress treatment, POD activity was significantly enhanced in *int51*, *int51/anac017-1,* and *anac017-2* in comparison to Col-0. Thus, the POD activities of the various genotypes matched the DAB staining well under the salt stress condition.

Because of the inverse accumulation of O_2_^•−^ and H_2_O_2_ in the mutants, we examined the enzymatic activity of SOD, which converts O_2_^•−^ into H_2_O_2_ [27,35]. As expected, total SOD (T-SOD) activity in leaves decreased in *int51* under the normal condition and markedly decreased in *int51*, *int51/anac017-1,* and *anac017-2* under salt stress as compared to Col-0 (Figure 6E). However, the transcript levels of all eight SOD genes were not significantly different in the mutants and Col-0 (Supplemental Figure S5B), implying that ANAC017 regulated SOD enzyme activity at the post-transcriptional level. In contrast, CAT activity, which converts H_2_O_2_ to H_2_O, did not show any difference between the mutants and Col-0 under both conditions (Figure 6F). Collectively, these results indicated that ANAC017 acted downstream of RCD1 to mediate ROS homeostasis, mainly by affecting the activities of T-SOD and POD.

### 2.6. ANAC017 Overexpression Transgenic Plants Are Hypersensitive to Salt Stress

**Figure 7:**
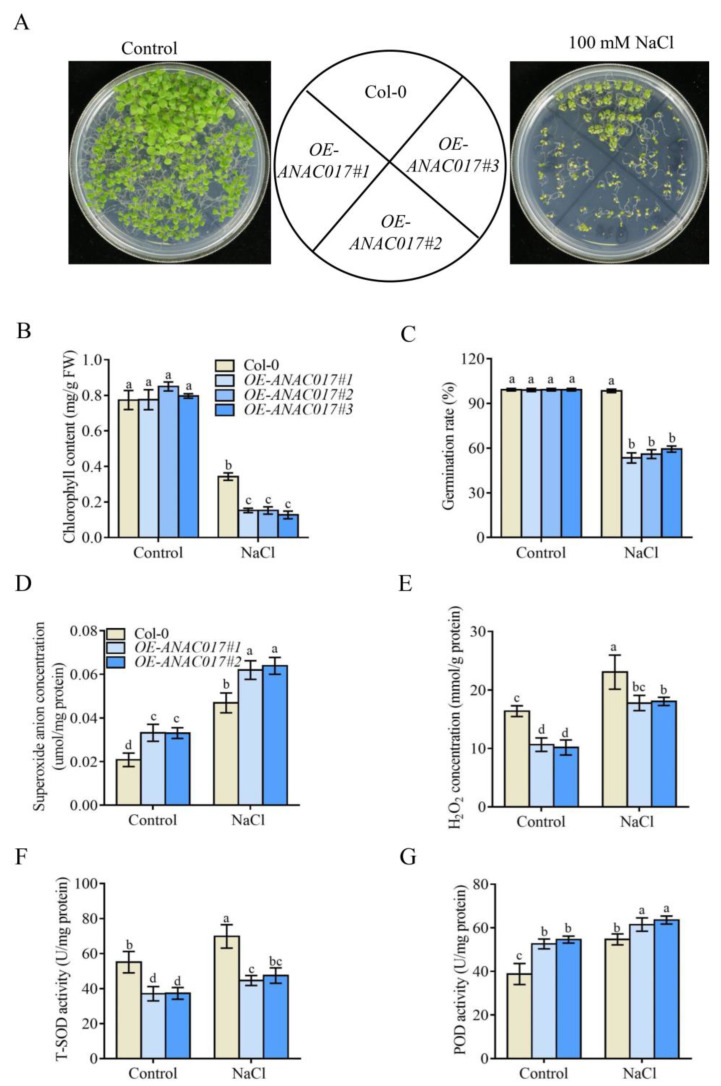
Overexpression of *ANAC017* results in hypersensitivity to salt stress. (**A**) The phenotype of fourteen-day-old Col-0, *OE-ANAC017#1*, *OE-ANAC017#2*, and *OE-ANAC017#3* grown on 1/2 MS with or without 100 mM NaCl. (**B**,**C**) Quantitative analysis of chlorophyll content (**B**) and germination rate (**C**) in WT and mutants. Data were collected from five biological replicates and presented as mean ± standard deviation. About 0.1 g seedlings were used for chlorophyll determination in each replicate. (**D**–**G**) Quantitative analysis of ROS molecules O_2_^•−^ (**D**) and H_2_O_2_ (**E**) and ROS-related enzyme activities of total SOD dismutase (**F**) and POD (**G**) in leaves of Col-0, *OE*-*ANAC017#1*, and *OE-ANAC017#2*. Seven-day-old seedlings grown on 1/2 MS medium were transferred to soil and grown for another two weeks before the treatment with or without 400 mM NaCl for four days. Data were collected from five biological replicates and presented as mean ± standard deviation. About 0.1 g rosette leaves were used in each replicate. Letters indicate significant differences at *p*-value < 0.05 based on one-way ANOVA and Tukey’s HSD test.

To further confirm the role of ANAC017 in salt stress response, we generated three independent transgenic lines overexpressing *ANAC017* (*OE-ANAC017*) in Col-0. The small, curled leaf and retarded growth phenotypes of these lines were consistent with previous reports (Figure 7A) [19,36]. The seed germination rate and chlorophyll content in *OE-ANAC017* lines were severely reduced compared to Col-0 under salt stress (Figure 7A–C). Similar to the *int51* mutant, the levels of O_2_^•−^ were increased, and those of H_2_O_2_ were reduced in two *OE-ANAC017* lines compared to Col-0 under both normal and salt stress conditions (Figure 7D,E). In contrast to T-SOD activity, which decreased in both *OE-ANAC017* lines, POD activity was increased (Figure 7F,G). Therefore, we conclude that ANAC017 plays a negative role in salt stress response by impairing the activities of antioxidant enzymes.

## 3. Discussion

### 3.1. RCD1 Positively Regulates Salt Stress Response by Repressing ANAC017 Transcriptional Activity

**Figure 8:**
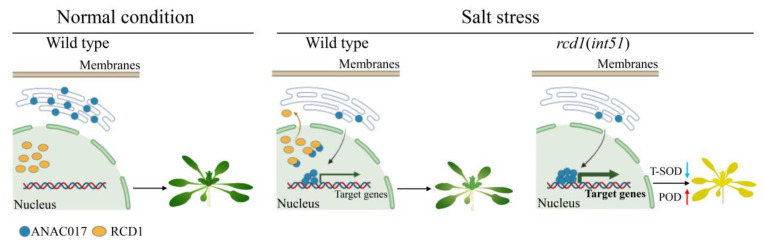
The proposed role of the RCD1-ANAC017 module in response to salt stress. Under normal growth conditions, the ANAC017 protein (blue solid dots) is predominantly located on the ER membrane, and the RCD1 protein (orange solid ovals) is located in the nucleus. When exposed to salt stress, in wild type, some RCD1 protein is exported from the nucleus to the cytoplasm, and a large amount of ANAC017 is released from the ER to the nucleus, where RCD1 interacts with ANAC017 and represses its transcriptional activity, resulting the expression of a few downstream target genes. In the absence of RCD1 (*int51* mutant) under salt stress, ANAC017 protein is released from the ER to the nucleus to activate numerous target genes, then reducing and enhancing the enzyme activities of T-SOD and POD, respectively, leading to developmental abnormalities and hypersensitivity to salt stress.

ANAC017 is an ER membrane-bound TF that is released by a rhomboid protease upon AA treatment to act in retrograde signaling in the nucleus [17]. In our study, a previously unknown function of ANAC017 in regulating salt stress response is uncovered (Figure 8). The loss-of-function *anac017-1* allele suppresses the salt hypersensitivity of an *rcd1* mutant, while the gain-of-function *anac017-2* mutant and *ANAC017* overexpression lines exhibit hypersensitivity to salt stress (Figure 1 and Figure 7A–C, Supplemental Figure S1), indicating that ANAC017 acts downstream of RCD1 and plays a negative role in salt tolerance.

RCD1 associates with multiple TFs, which may account for its pleiotropic functions in plants, and the interaction between RCD1 and ANAC017 was established by pull-down assays [10,11,15], although these two proteins are located in the nucleus and ER membrane, respectively, under unstressed condition. Similar to AA treatment, salt stress induces the cytoplasm-to-nucleus translocation of ANAC017 (Figure 2A,B), which is mimicked by the truncated ANAC017-ΔTM, and the interaction between RCD1 and ANAC017-ΔTM is observed in BiFC assays (Figure 3B). In *Chrysanthemum*, CmRCD1 is reported to physically interact with B-BOX DOMAIN PROTEIN 8 (CmBBX8) and reduce CmBBX8′s ability to bind the promoter of FLOWERING LOCUS T-LIKE 1 (CmFTL1) [37]. Likewise, ANAC017-ΔTM activates the expression of *AOX1a*, while RCD1 markedly reduces the transcriptional activity via its interaction with ANAC017-ΔTM in the nucleus (Figure 3C,D). Consistently, our RNA-seq analysis reveals that 282 out of the 743 up-regulated genes in *int51* show substantially decreased expression levels in *int51/anac017-1* (Figure 4C, Supplemental Data Set S1). Besides, 95 up-regulated and 69 down-regulated genes are shared by *int51* and *anac017-2*. These results indicate that ANAC017 is active in the absence of RCD1. In addition, 82 candidate genes containing the ANAC017-binding motif are likely direct targets of the RCD1-ANAC017 module, including the previously reported genes *AOX1a* and *UGT74E2* (Supplemental Figure S3, Supplemental Data Set S2), supporting the notion that *anac17-1* is epistatic to *int51* in both retrograde signaling and salt stress response.

### 3.2. ANAC017 Confers Salt Hypersensitivity through Affecting ROS Homeostasis

Overaccumulation of ROS causes oxidative stress that can lead to damage to organelles and even cell death in plants [26,38,39]. The fact that many DEGs in *int51* and *anac017-2* are involved in oxidation-reduction processes (Figure 5A,B) suggests that the RCD1-ANAC017 module may impact ROS homeostasis. Indeed, under salt stress treatment, *int51*, *anac017-2*, and *OE-ANAC017* transgenic lines produce more O_2_^•−^, but less H_2_O_2_, compared to Col-0, while *anac017-1* suppresses the abnormal levels of O_2_^•−^ and H_2_O_2_ in *int51* (Figure 6C,D and Figure 7D,E), indicating that RCD1 maintains ROS homeostasis under salt stress through repressing ANAC017. In addition, salt-induced T-SOD enzyme activity is severely impaired in *int51*, *anac017-2*, and *OE-ANAC017* (Figure 6E and Figure 7F). O_2_^•−^ is usually the first product generated from O_2_ consumption in plant organelles and further triggers the formation of other ROS, such as OH^•^ and ^1^O_2_. The metalloenzyme SOD is the most effective dismutase reducing O_2_^•−^ to H_2_O_2_. Extensive genetic evidence in different species has demonstrated that reduction or disruption of SOD enzymatic activities leads to severe developmental defects, and overexpression of SOD genes results in enhanced tolerance when plants are subjected to abiotic stresses [40,41,42,43,44]. In *Arabidopsis*, eight SODs are classified into three groups, including three copper/zinc (Cu/Zn)-SODs, two manganese (Mn)-SODs, and three iron (Fe)-SODs, according to their metal cofactors [28,45,46]. The transcript levels of these SOD genes are not significantly different in the mutants and Col-0 (Supplemental Figure S5B), implying that RCD1 and ANAC017 regulate SOD enzyme activity at the post-transcriptional level. Collectively, our data support the hypothesis that RCD1 interacts with ANAC017 and inhibits its transcriptional activation activity. Constitutive or excessive function of ANAC017 disrupts ROS homeostasis by reducing T-SOD activity, perhaps indirectly and especially under salt stress (Figure 8).

## 4. Materials and Methods

### 4.1. Plant Materials and Growth Conditions

The *Arabidopsis thaliana* mutants and transgenic plants used in this study were all in the Col-0 background. *anac017-1* (SALK_022174), *anac017-2* (SALK_044777), and *int51* (*rcd1-5*) were described before [17,33], and the *int51 anac017-1* double mutant was obtained by crosses. *35S::GFP-ANAC017*(*OE-ANAC017*) transgenic plants were generated by cloning the full-length coding sequence of *ANAC017* into the pEG101 overexpression vector containing N-terminal GFP tag and transforming Col-0 plants with the plasmid via Agrobacterium-mediated floral dipping. Individual transgenic lines of *OE-ANAC017* were selected by Basta resistance. Seeds were plated on the 1/2 MS (Murashige and Skoog Basal Medium) containing 1% sucrose and 0.8% agar, incubated for 3 days at 4 °C and then transferred to 22 °C climate-controlled chambers under 16 h light/8 h dark cycles. For the seed germination assays, the emergence of green cotyledons was used as the indicator of germination. Seven-day-old seedlings were transferred to soil for other experiments. For the treatments of AA (antimycin A) and NaCl on *N. benthamiana*, the leaves of *N. benthamiana* were sprayed with or without 50 µM AA solution, and the plants growing soil were supplied with or without 200 mM NaCl solution. For NaCl induction assays, transgenic plants harboring the fused GFP-ANAC017 protein grown on the 1/2 MS plates for seven days and were transferred to 1/2 MS medium with or without 100 mM NaCl. The primers used in this study are listed in Supplemental Data Set S3.

### 4.2. Chlorophyll Content Assay

Total chlorophyll was extracted from 14-day-old seedlings of Col-0, *int51*, *int51 anac017-1*, *anac017-1*, *anac017-2,* and *OE-ANAC017*, respectively, and was measured according to procedures described before [47].

### 4.3. Subcellular Localization of RCD1 and ANAC017 in Arabidopsis protoplasts and N. benthamiana Leaves

The full-length coding sequences of *RCD1* and *ANAC017* from Col-0 were cloned into the pGD-eGFP vector [48] to generate *pGD-RCD1-eGFP* and *pGD-eGFP-ANAC017*, respectively. The plasmids were transformed into the Agrobacterium strain GV3101 and the Agrobacteria in medium (10 mM MES, 200 μM AS, 10 mM MgCl_2_) were transfected into the *Arabidopsis* protoplasts using polyethylene glycol (PEG)-mediated transformation [49] or introduced into 4-5-week-old *N. benthamiana* leaves via syringe with infiltration medium (10 mM MES, 200 μM AS, 10 mM MgCl_2_). *Arabidopsis* protoplasts were incubated in the dark for 16 h at 23 °C and *N. benthamiana* were grown for 48–72h before GFP and RFP signals were observed using confocal laser scanning microscopy with the excitation wavelengths of 488 and 532 nm, respectively. The ER marker ER-rk *CD3-959* [50] and the nuclear marker H2B-mCherry were used for colocalization analysis.

### 4.4. Yeast Two-Hybrid Assay

The full-length coding sequences of *RCD1* and *ANAC017* were cloned into the *pGBKT7* and *pGADT7* vectors (Clontech, Mountain View, CA, USA), respectively, and these constructs were transformed to the yeast strain Y2HGold (Clontech, Mountain View, CA, USA) using the LiAc/TE method. The yeast cells were grown on the medium supplemented with SD-Leu-Trp-His or SD-Ade-Leu-Trp-His.

### 4.5. Bimolecular Fluorescence Complementation Assays (BiFC) in N. benthamiana Leaves

The coding sequences of *RCD1* and *ANAC017ΔTM* from Col-0 were cloned into pPVYNE and pPVYCE vectors, respectively, to generate *p35S::RCD1-N-VENUS* and *p35S:: ANAC017ΔTM-C-VENUS*. The plasmids were transformed into the Agrobacterium strain GV3101 and the *Agrobacteria* in medium (10 mM MES, 200 μM AS, 10 mM MgCl_2_) were introduced into 4-5-week-old *N. benthamiana* leaves via syringe infiltration. After plants were grown for another 48–72 h, the VENUS and mCherry signals were observed using confocal laser scanning microscopy with excitation wavelengths of 515 and 543 nm, respectively.

### 4.6. Dual Luciferase Transient Activation Experiment in N. benthamiana

The coding sequences of *RCD1* and *ANAC017ΔTM* from Col-0 were cloned into the pGreen II 62-SK vector [51]. A 2 kb promoter of the *AOX1a* gene containing two ANAC017 binding sites was amplified and introduced into the reporter vector pGreen II 0800-LUC [51] to generate *pGreen II 0800-AOX1a-LUC*. The *Renilla LUC* gene driven by the *35S* promoter (*pGreen II 0800-LUC*) was used as the internal control. The reporter construct, together with *pSK-ANAC017ΔTM* and/or *pSK-RCD1*, was transformed into the Agrobacterium strain GV3101 and the Agrobacteria in medium (10 mM MES, 200 μM AS, 10 mM MgCl_2_) were co-transformed into *N. benthamiana* leaves. Firefly and Renilla luciferase activity was measured using the dual luciferase reporter assay system (Promega, Madison, WI, USA) according to the manufacturer’s instructions.

### 4.7. Transcriptome Sequencing and Data Analysis

Total RNA was extracted from each genotype using the TRIzol^®^ Reagent (Invitrogen, Waltham, MA, USA). RNA-seq libraries were constructed and sequenced on an Illumina Hiseq ×10 platform to generate pair-end reads of 150 bp in length by Berry Genomics Co., Ltd. (Beijing, China). The raw RNA-seq data were analyzed using pRNASeqTools (https://github.com/grubbybio, accessed on 4 October 2022). Differentially expressed genes (DEGs) among different samples were identified with the cut-off of fold-change > 2 (for hyper-DEGs) or fold-change < 0.5 (for hypo-DEGs) and *p* < 0.05. Gene Ontology (GO) enrichment analysis and box plot diagrams were performed using the R package ‘clusterProfiler’. The volcano plots, Venn diagrams, and heatmaps were generated with TBtools software [52].

### 4.8. Staining and Measurement of H_2_O_2_ and O_2_^•−^

The accumulation of H_2_O_2_ and O_2_^•−^ in leaves of each genotype was visualized by staining with DAB and NBT, respectively. The removal of chlorophyll was done by incubating the leaves in absolute ethanol in a boiling water bath, as described by [53]. The quantitative measurements of H_2_O_2_ and O_2_^•−^ contents in plant leaves were performed with a commercial hydrogen peroxide assay kit (Beijing Solarbio Science and Technology Co., Ltd., Beijing, China) and a superoxide anion assay kit (Beijing Solarbio Science and Technology Co., Ltd., Beijing, China), respectively.

### 4.9. Measurement of the Activities of Antioxidant Enzymes

Plant tissues were ground in liquid nitrogen and mixed with 0.9 mL solution containing 10 mM phosphate buffer (pH 7.4). The suspension was centrifuged at 3500 rpm for 10 min at 4 °C, and the supernatant was collected for activity assays for T-SOD, POD, and CAT with commercial assay kits (Nanjing Jiancheng Bioengineering Institute, Nanjing, China).

## Data Availability

The data reported in this paper have been deposited in NCBI Sequence Read Archive (SRA) database (https://submit.ncbi.nlm.nih.gov/subs/sra/) with the accession no. PRJNA883985 and accessed on 4 October 2022.

## Supplementary Materials

The following supporting information can be downloaded at: https://www.mdpi.com/article/10.3390/ijms24129793/s1.
