# Supplementary material for: RCD1 Promotes Salt Stress Tolerance in Arabidopsis by Repressing ANAC017 Activity
Source: Int J Mol Sci. 2023 Jun 6;24(12):9793. doi: 10.3390/ijms24129793 (PMC10298584; doi:10.3390/ijms24129793)
Supplement: Supplementary file 1 [file ijms-24-09793-s001.zip › Supplemental Data Fugures 1-5.pdf]

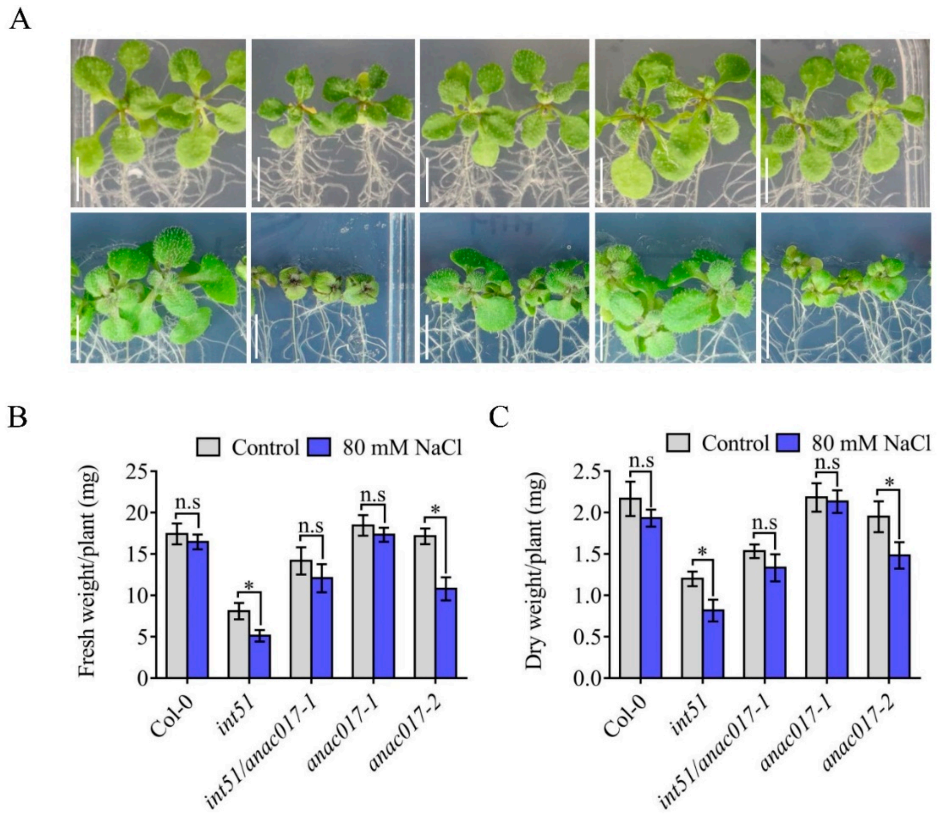

Figure S1. Loss of function in *ANAC017* alleviates the salt sensitivity of *int51*.
(A) The phenotype of Col-0, *int51*, *int51/anac017-1*, *anac017-1*, and *anac017-2* seedlings grown vertically in response to the low salt stress. 5-day-old seedlings were transferred to 1/2 MS medium with or without 80 mM NaCl and grown for another 12 days. Bar scale, 1 cm. (B-C) Quantitative analysis of fresh weight (B) and dry weight (C) per plant in (A). Data were collected from three biological replicates (n =12 biologically independent plants) and presented as mean ± standard deviation. n.s, not significant. Asterisks indicated significant differences as determined by two-sided Student's *t*-test. *, *p*-value <0.01.

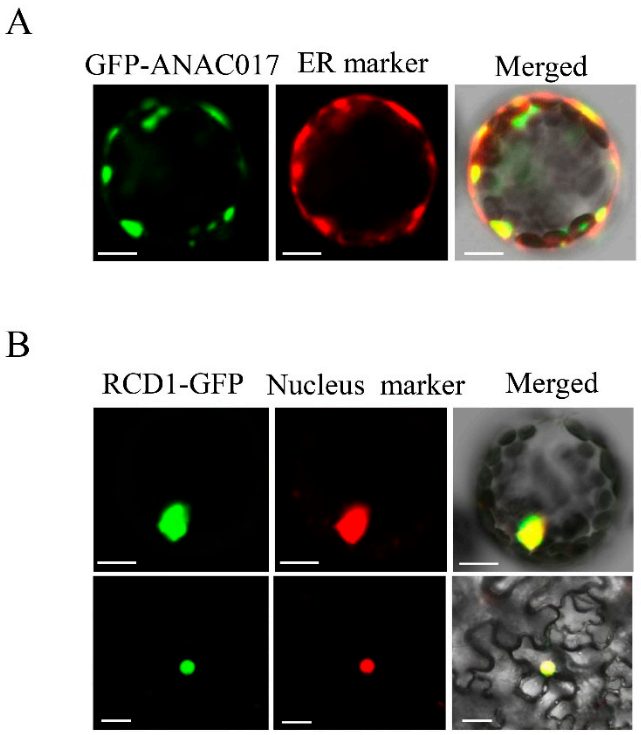

Figure S2. Subcellular localization of ANAC017 and RCD1.
(A) The subcellular localization of co-expressed *35S::GFP-ANAC017* and an ER marker (ER-rk *CD3-959*) in *Arabidopsis* protoplasts. (B) The subcellular localization of co-expressed *35S::RCD1-GFP* and a nucleus marker (H2B-mCherry) in *Arabidopsis* protoplasts (top) and *N. benthamiana* leaves (bottom), respectively. Bar scale, 20 μm.

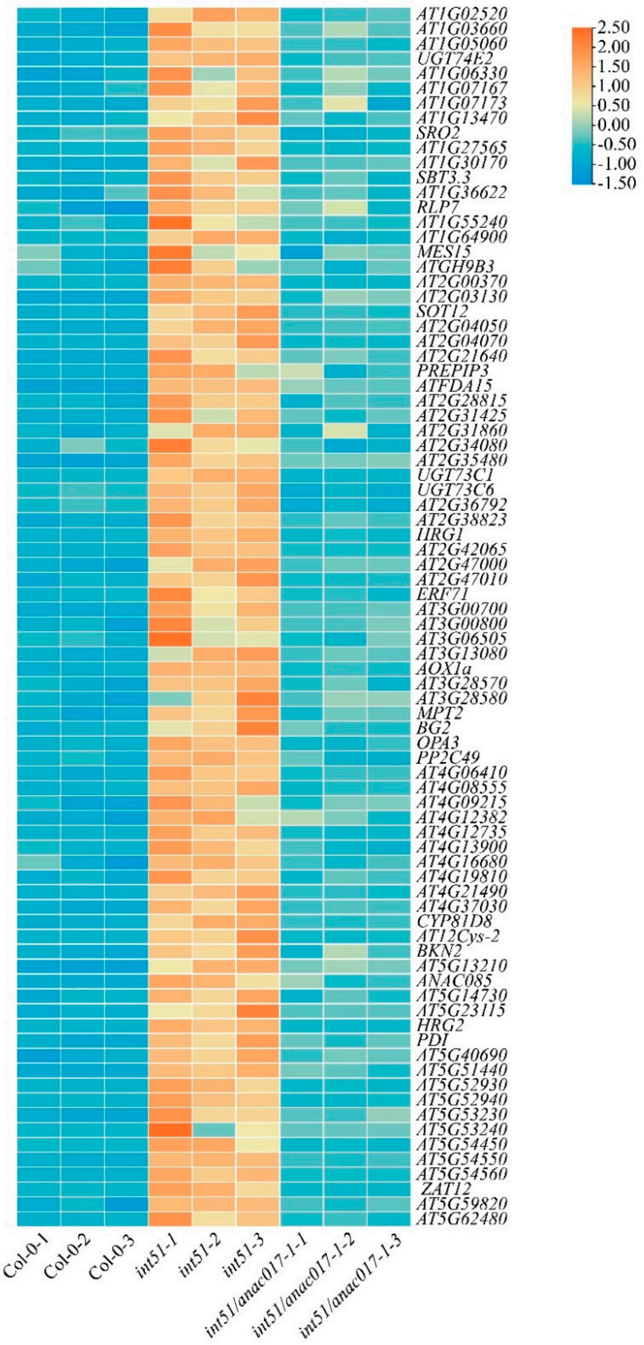

Figure S3. RNA-seq analysis of *int51*, *int51/anac017-1* for differentially expressed genes by comparing with Col-0.

Heatmap showing the relative transcript abundance of 82 candidate genes containing the mitochondrial dysfunction motif (MDM) in WT and the mutants. The colors from blue to red indicate the gradually increased relative abundance. The X axis represents each genotype with three biological replicates, and the Y axis represents the genes. Three

biological replicates are shown for each genotype.

A

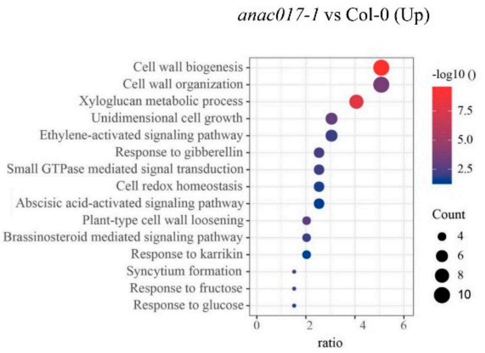

B

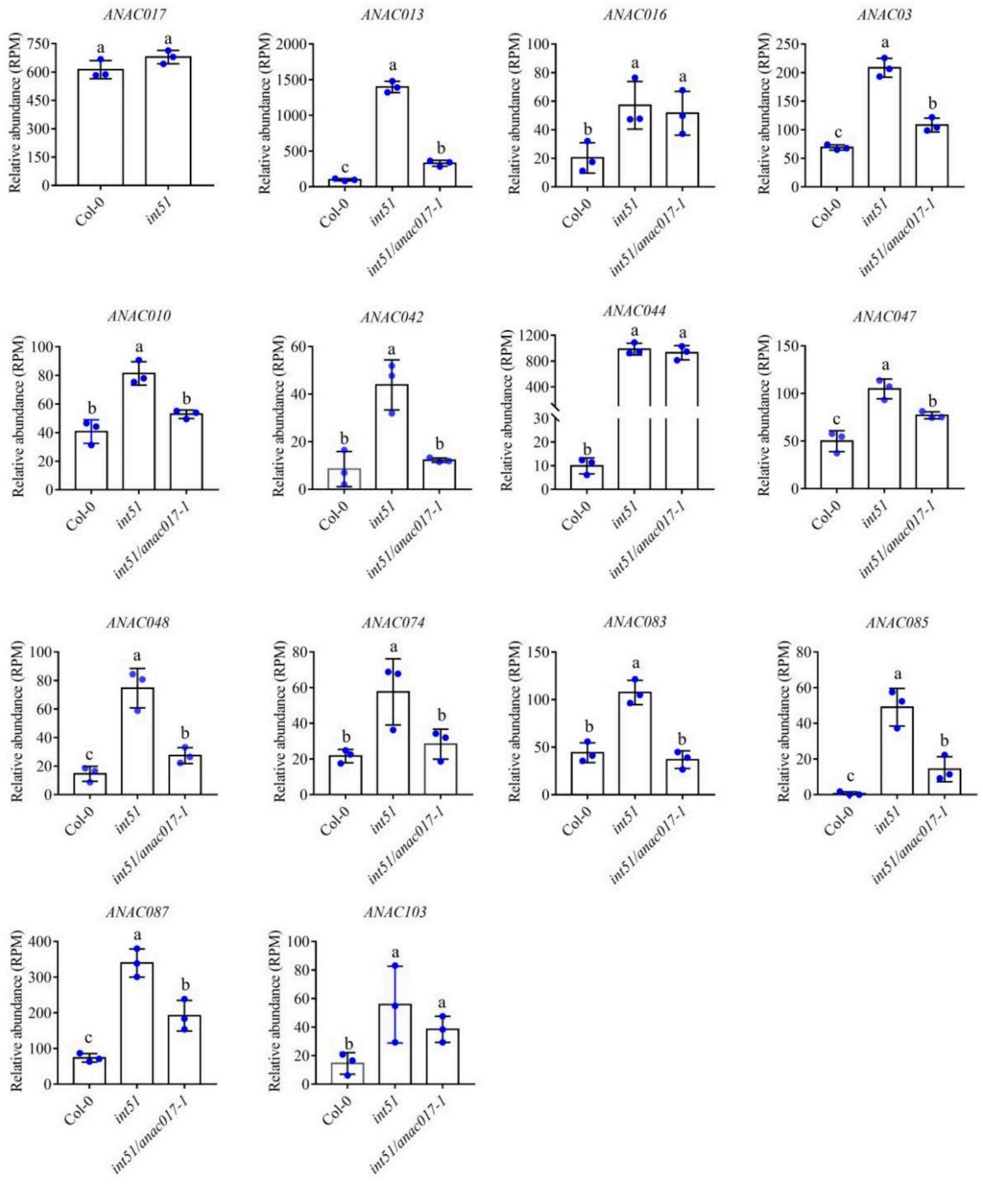

Figure S4. Differentially expressed genes in *anac017-1* compared to Col-0.
(A) GO enrichment analysis of the up-regulated genes in *anac017-1* compared to Col-0. (B) The relative expression of *ANAC* family genes in the *int51* mutant and the *int51/anac017-1* double mutant compared to Col-0. Data presented as mean ± standard deviation. Letters indicate significant differences at *p*-value < 0.05 based on one-way ANOVA and Tukey's HSD test.

A

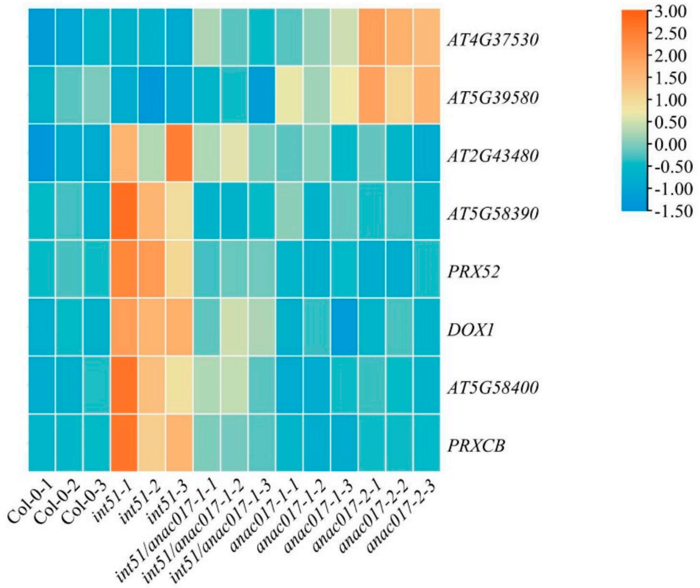

B

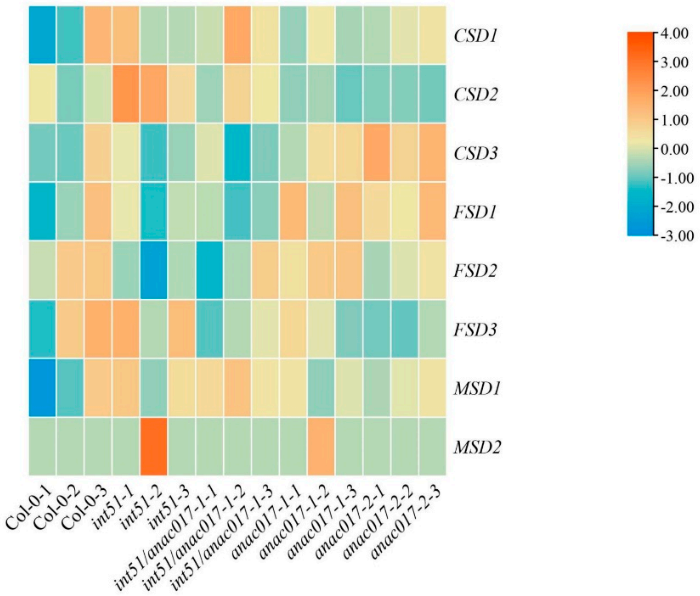

Figure S5. Heatmap showing the expression of peroxidase and superoxide dismutase family genes in Col-0 and mutants.

Relative expression levels of genes encoding peroxidase (A) and superoxide dismutase (B) are shown. The colors from blue to red represent gradually increased relative abundance. The genotypes, each with three replicates, are labeled at the bottom and the gene names (or identifiers) are listed on the right.
